# Positive peritoneal cytology in early-stage endometrial cancer does not influence prognosis

**DOI:** 10.1038/sj.bjc.6602035

**Published:** 2004-07-13

**Authors:** P-M Tebeu, Y Popowski, H M Verkooijen, C Bouchardy, F Ludicke, M Usel, A L Major

**Affiliations:** 1 Fondation pour Recherches Médicales, University of Geneva, Switzerland; 2 Department of Obstetrics and Gynaecology, Yaounde University Hospitals, Cameroon; 3 Department of Radiation Oncology, Geneva University Hospitals, Switzerland; 4 Geneva Cancer Registry, Institute for Social and Preventive Medicine, Geneva University, Switzerland; 5 Department of Obstetrics and Gynaecology, Geneva University Hospitals, Switzerland

**Keywords:** endometrial cancer, peritoneal cytology, survival, stage, prognosis

## Abstract

The aim of this study was to assess the prognostic importance of positive peritoneal cytology in early-stage endometrial cancer. All 278 stage I and 53 stage IIIA (without cervical involvement) endometrial cancer patients operated between 1980 and 1996, recorded at the Geneva Cancer registry, were included. Stage IIIA cancers were recategorised into ‘cytological’ stage IIIA (positive peritoneal cytology alone, *n*=33) and ‘histological’ stage IIIA (serosal or adnexal infiltration, *n*=20). Survival rates were analysed by Kaplan–Meier method and compared using log-rank test. The prognostic importance of cytology was analysed using a Cox model, accounting for other prognostic factors. The 5-year disease-specific survival of cytological stage IIIA cancer was similar to stage I (91 *vs* 92%) and better than histological stage IIIA cancer (50%, *P*<0.001). After adjustment for age, myometrial invasion, differentiation and radiotherapy, cytological stage IIIA patients were still at similar risk to die from endometrial cancer compared to stage I patients (hazard ratio (HR) 0.7, 95% confidence interval (CI): 0.18–2.3), while histological stage IIIA patients were at a four-fold increased risk to die from their disease (HR 4.2, 95% CI: 1.7–10.3). This population-based study shows that positive peritoneal cytology in itself has no impact on survival of patients with localised endometrial cancer. Based on the present and previous studies, FIGO (Fédération Internationale de Gynécologie et d'Obstétrique) might consider reviewing its classification system.

In 1988, FIGO (Fédération Internationale de Gynécologie et d'Obstétrique) introduced a new classification system for staging endometrial cancer, based mainly on surgical findings (Grady and Ernster, 1996; Burke *et al*, 1997). In addition to pathological extension, peritoneal cytology became an important determinant in this new classification system. In particular, stage I patients (i.e. endometrial cancer confined to the uterus) and stage II patients (i.e. endometrial cancer invading the cervix) who demonstrated positive peritoneal cytology were upstaged to stage IIIA (Konski *et al*, 1988). Stage IIIA therefore includes at the same time patients with positive peritoneal cytology alone and patients with macroscopic/histological invasion of serosa or adnexal tissues, suggesting that these patients have similar prognosis.

Since then, some studies suggested that peritoneal cytology is not an important prognostic factor (Grimshaw *et al*, 1990; Kadar *et al*, 1992; Preyer *et al*, 2002; Kasamatsu *et al*, 2003). In a recent study, including endometrial cancer patients treated with surgery and radiotherapy, we showed that patients classified as stage IIIA because of positive peritoneal cytology had the same survival as stage I endometrial cancer patients (Tebeu *et al*, 2003). However, as this hospital-based study included only patients who received radiotherapy, we could not rule out that the lack of survival difference was either real or due to the benefit of adjuvant radiotherapy. In order to study the prognostic importance of peritoneal cytology, independent of radiation therapy, we extended our previous study population to nonirradiated patients, using population-based data.

## MATERIALS AND METHODS

The current study was performed with information of the population based cancer registry of the Swiss canton of Geneva (approximately 420 000 inhabitants). The registry records information on all incident cases of malignant neoplasms occurring in the canton among the resident population. Information is collected from various sources (i.e. pathology reports, medical files from public hospitals and private physicians), and is considered very accurate, confirmed by the very low percentage (<1%) of cases recorded from death certificates only (Bouchardy, 1997).

The registry systematically records data on sociodemographic status, diagnostic circumstances, modalities of diagnostic assessment, tumour characteristics coded according to the International Classification of Diseases for Oncology WHO (1976), stage of the disease at diagnosis, treatment during the first 6 months after diagnosis, survival status and cause of death.

For this particular study, we opened the clinical files to obtain additional data on peritoneal cytological assessment, degree of myometrial invasion and detail on the type of surgery and adjuvant radiotherapy. We recoded the stages according to the 1988 FIGO staging system: stage I, tumour confined to uterus; stage II, tumour invading cervix; stage IIIA, tumour associated with positive peritoneal cytology or with macroscopic or histological involvement of serosa or adnexa; and stage IIIB+, tumour invading vagina, mucosa of bladder/bowel, regional lymph node or with distant metastases. For the purpose of the present study, stage IIIA cancers were further categorised as: ‘cytological’ stage IIIA defined as patients with stage IIIA endometrial cancer based on positive peritoneal cytology only and ‘histological’ stage IIIA defined as stage IIIA patients with histological or macroscopic infiltration of serosa or adnexal tissues.

Other variables of interest were: age at diagnosis (<50, 50–69, ⩾70 years), period of diagnosis (1980–1987, 1988–1996), differentiation (good, moderate, poor, unknown), degree of myometrial invasion (invasion <50%, invasion ⩾50%), type of surgery (hysterectomy and oophorosalpingectomy with and without lymphadenectomy) and type of radiotherapy (no radiotherapy, external radiotherapy with brachytherapy, external radiotherapy only, brachytherapy only). For the purpose of the present study, patients were regrouped as ‘radiotherapy group’ defined as cases with any adjuvant radiotherapy and ‘no radiotherapy group’ defined as cases with no adjuvant radiotherapy.

Data on survival and follow-up were derived from the Geneva cancer registry and included vital status, date of death or departure from the canton (regularly and systematically obtained from the Cantonal Population Office) and cause of death (retrieved from medical files).

### Patients

We considered all resident women diagnosed with endometrial cancer between 1980 and 1996 in the Swiss canton of Geneva (*n*=731). We excluded patients who were not treated surgically (*n*=87), patients with uterine sarcomas (*n*=24), patients with other malignancies occurring within 5 years prior to or within 6 months from endometrial cancer diagnosis (*n*=74) and patients with missing information on histology or stage (*n*=7). We also excluded patients with stage I endometrial cancer without peritoneal cytological assessment (*n*=139). Of the 400 remaining patients, 278 were diagnosed as stage I, 29 as stage II, 38 as cytological stage IIIA (33 without and five with cervical involvement), 20 as histological stage IIIA and 35 as stage IIIB+. For this study, only patients with stage I, cytological stage IIIA without cervical involvement and histological stage IIIA endometrial cancer were included (*n*=331).

Peritoneal washing was performed by either collecting liquid present in the peritoneal cavity or by rinsing the cavity with 100 cm^3^ of physiological saline. The liquid was centrifuged and assessed for the presence of malignant cells. A patient was considered to have positive peritoneal cytology if adenocarcinoma cells were detected, regardless of the number of cancer cells. Surgical treatment included hysterectomy generally including oophorosalpingectomy. Lymphadenectomy was not routinely performed.

### Statistical analyses

The 5-year disease-specific survival rates were calculated considering death from endometrial cancer only by Kaplan–Meier method and compared by nonparametric survival analyses using log-rank test. The effect of peritoneal cytology on endometrial cancer mortality was analysed by multivariate Cox's proportional-hazards modelling, taking into account other variables significantly linked to survival. Analyses were performed using SPSS (Hull and Nie, 1995). Differences were considered statistically significant at *P*<0.05. Additional subgroup analyses were performed among patients with and without additional radiotherapy.

## RESULTS

Patient and tumour characteristics and treatment modalities of the stage I (*n*=278), cytological stage IIIA without cervical involvement (*n*=33) and histological stage IIIA (*n*=20) patients are described in Table 1

**Table 1:** Characteristics of endometrial cancer patients according to stage at diagnosis

	**Stage at diagnosis**			
	**I**	**‘Cytological’ IIIA^a^**	**‘Histological’ IIIA**	**Total**
	***n*=278**	***n*=33**	***n*=20**	***n*=331**
**Characteristics**	***n* (%)**	***n* (%)**	***n* (%)**	***n* (%)**
Mean age (range)	64.8 (33–90)	65.8 (48–85)	68.0 (48–83)	65.1 (33–91)
*Age category (years)*				
<50	18 (7)	1 (3)	1 (5)	20 (6)
50–69	170 (61)	21 (64)	12 (60)	203 (61)
70+	90 (32)	11 (33)	7 (35)	108 (33)
				
*Period of diagnosis*				
1980*–*87	129 (46)	9 (27)	7 (35)	145 (44)
1988*–*96	149 (54)	24 (73)	13 (65)	186 (56)
				
*Invasion of myometrium*				
<50%	211 (76)	18 (55)	4 (20)	233 (70)
⩾50%	63 (23)	15 (45)	16 (80)	94 (28)
Unknown	4 (1)	—	—	4 (1)
				
*Differentiation*				
Good	176 (63)	15 (45)	7 (35)	198 (60)
Moderate	63 (23)	12 (36)	5 (25)	80 (24)
Poor/undifferentiated	28 (10)	4 (12)	7 (35)	39 (12)
Unknown	11 (4)	2 (6)	1 (5)	14 (4)
				
*Surgical procedure*				
Hysterectomy	12 (4)	—	—	12 (4)
Hysterectomy and annexectomy^b^	247 (89)	33 (100)	19 (95)	299 (90)
Hysterectomy, annexectomy^b^ and lymphadenectomy	4 (1)	—	1 (5)	5 (2)
Unspecified type of surgery	15 (5)	—	—	15 (5)
				
*Radiotherapy*				
None	149 (54)	6 (18)	5^c^ (25)	160 (48)
External and brachy	46 (17)	14 (42)	11 (55)	71 (22)
External only	10 (4)	9 (27)	4 (20)	23 (7)
Brachy only	72 (26)	4 (12)	—	76 (23)
Unknown type	1 (<1)	—	—	1 (<1)

a Excluding patients with cervical invasion.

b Nine patients had unilateral annexectomy and seven had previous unilateral annexectomy because of cysts or extrauterine gravities. One 41-year-old patient had only one ovary removed and the other left in place because of her young age and in one patient the reason was unknown.

c One of these patients received adjuvant chemotherapy.

. Compared to stage I patients, women with histological stage IIIA cancer were slightly older, had less well-differentiated tumours and had more often myometrial invasion of more than 50%. Cytological stage IIIA tumours were less differentiated and invaded the myometrium to a deeper extent. Overall, 171 (52%) women underwent adjuvant radiotherapy. In all, 46% (*n*=129) of stage I patients had radiotherapy compared to 83% (*n*=27) of cytological stage IIIA and 75% (*n*=15) of histological stage IIIA patients.

Figure 1

**Figure 1:**
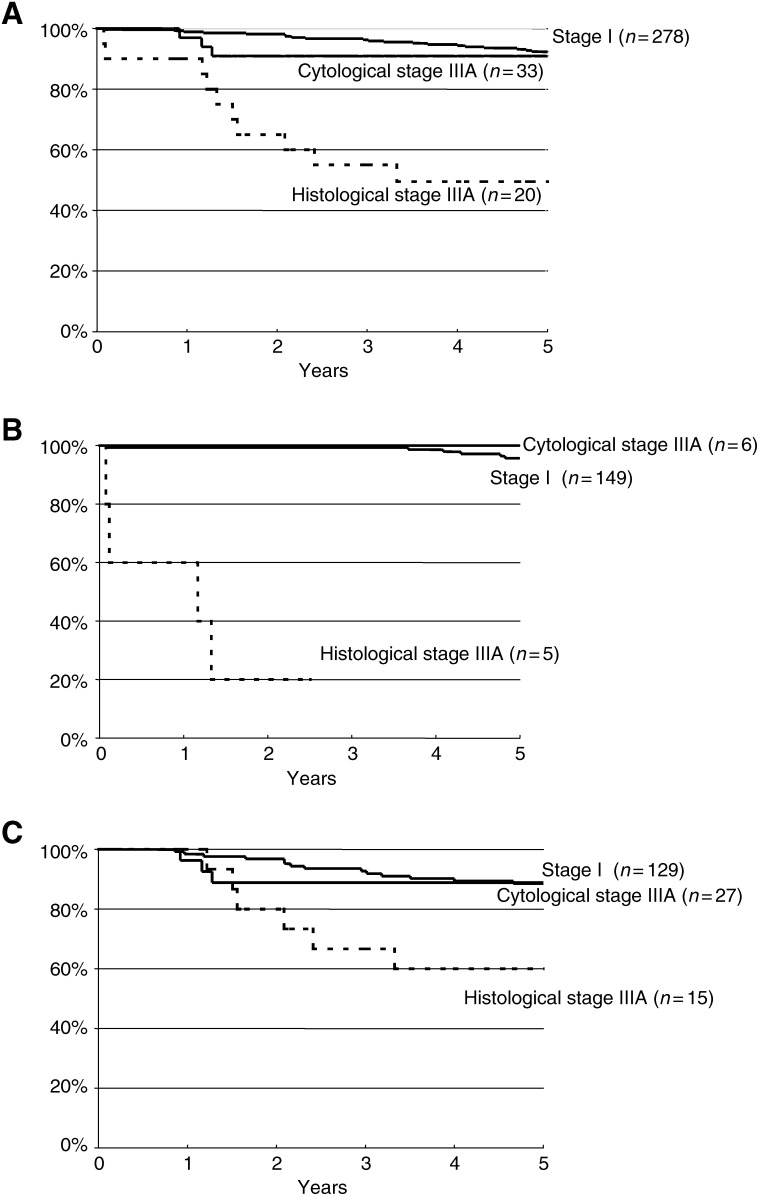
Disease-specific survival curves of endometrial cancer according to stage – stage I, cytological stage IIIA (without cervical invasion), histological stage IIIA – for all patients (**A**), patients who did not receive radiotherapy (**B**) and patients who received radiotherapy (**C**).

presents the 5-year disease-specific survival curves according to stage. For all patients combined (with and without adjuvant radiotherapy), survival for stage I endometrial cancer (92.3%) was comparable to that of cytological stage IIIA endometrial cancer (90.9%). In contrast, patients with histological stage IIIA cancer had a significantly worse survival (49.5%, *P*<0.001). When separately analysing patients with and without adjuvant radiotherapy, similar results were observed (Figure 1B and C). There was still no disease-specific survival difference between stage I and cytological stage IIIA cancer patients and a lower survival rate for histological stage IIIA patients.

Table 2

**Table 2:** Disease-specific survival and hazard ratios for death from endometrial cancer according to stage

	***n***	**Endometrial cancer deaths (*n*)**	**5-year disease-specific survival (%)**	**Crude HR^a^ (95% CI)**	**Adjusted HR^b^ (95% CI)**
*Stage*					
Stage I	278	20	92.3	1^c^	1^c^
Cytological stage IIIA^d^	33	3	90.9	1.3 (0.4–4.3)	0.74 (0.2–2.3)
Histological stage IIIA	20	10	49.5	10.5 ***(4.8–22.7)	4.2 **(1.7–10.3)
					
*Myometrial invasion*					
<50%^e^	236			1^c^	1^c^
⩾50%	95			3.5 ***(1.7–7.1)	1.8 (0.8–3.9)
					
*Differentiation*					
Good	198			1^c^	1^c^
Moderate	80			5.3 ***(2.0–14.1)	4.6 **(1.7–12.3)
Poor	39			15.0 ***(5.7–39.0)	9.8 ***(3.6–26.8)
Unknown	14			2.7 (0.3–22.0)	2.8 (0.3–23.4)
					
*Radiotherapy*					
No	160			1^c^	1^c^
Yes	171			2.7 *(1.3–5.8)	1.7 (0.7–4.0)

HR=hazard ratio; CI=confidence interval.

* *P*<0.05,

** *P*<0.01,

*** *P*<0.001.

a Only adjusted for age (continuous).

b Alternately adjusted for age (continuous), stage, tumour differentiation, myometrial invasion and radiotherapy.

c Reference category.

d Excluding patients with cervical invasion.

e Including four patients (<1%) with unknown degree of myometrial invasion.

presents the 5-year disease-specific survival rates and the adjusted risks (hazard ratio's) of endometrial cancer mortality for stage, myometrial invasion, differentiation and radiotherapy. After adjustment for age, tumour differentiation, myometrial invasion and radiotherapy, cytological stage IIIA patients were not at an increased risk to die from endometrial cancer compared to patients with stage I disease (hazard ratio (HR) 0.7, 95% confidence interval (CI): 0.2–2.3). Histological stage IIIA patients, however, had a four-fold increased risk to die from their disease (HR 4.2, 95% CI: 1.7–10.3). Also, moderate or poor tumour differentiation significantly and independently increased the risk to die of endometrial cancer (adjusted HR 4.6, 95% CI: 1.7–12.3, and 9.8, 95% CI: 3.6–26.8, respectively). Myometrial invasion and radiotherapy were not significantly and independently linked to endometrial cancer mortality.

A total of 15 patients had clear cell carcinoma (*n*=7) or papillary serous carcinoma (*n*=8). Three (20%) were categorised as cytological stage IIIa. Excluding these 15 patients from the analyses did not significantly modify the results.

## DISCUSSION

In a recent publication, we have shown that positive peritoneal cytology was not a prognostic factor for patients with early-stage endometrial cancer treated with radiotherapy (Tebeu *et al*, 2003). We could, however, not rule out that the high survival of cytological stage IIIA patients was due to the use of radiotherapy, because in this study we included only irradiated patients. We therefore decided to extend our study population to all operated women, treated or not by radiotherapy. The present study confirms our earlier findings and shows that patients with ‘cytological stage IIIA’ endometrial cancer have the same prognosis as stage I patients, also after adjusting for other important prognostic factors, such as myometrial invasion, grade and, in particular, use of radiotherapy. In addition, similar prognosis of women with stage I and cytological stage IIIA endometrial cancer was observed among women with and without radiotherapy.

Based on several studies showing a relation between positive peritoneal cytology and unfavourable prognostic factors (lymph node invasion, grade, myometrial invasion, etc.) (Creasman *et al*, 1981; Ide, 1984), FIGO (2000) decided to incorporate cytology in its classification system, upgrading stage I and stage II patients with positive peritoneal cytology to stage IIIA (Creasman *et al*, 1987).

Since then, three studies confirmed the importance of peritoneal cytology by showing that patients with early-stage endometrial cancer with positive peritoneal cytology had worse survival rates than patients with early-stage endometrial cancer with negative peritoneal cytology (Harouny *et al*, 1988; Morrow *et al*, 1991; Obermair *et al*, 2001). As these studies classified patients according to the clinical staging system (AJCC, 2002) (based on hysterometry and physical examination), other important prognostic factors like myometrial invasion (Creasman *et al*, 1987) were not taken into account. As a result, positive cytology appeared to be an independent prognostic factor, while in fact it is more likely a consequence of aggressive or more advanced cancer. This goes along with our own findings, where tumours with deeper invasion and poor differentiation were associated more often with positive peritoneal cytology. In addition, these studies did not present the number of patients with cervical involvement and an unequal distribution of this important prognostic parameter between patients with and without positive cytology might have influenced prognosis.

Five publications used the new FIGO surgical staging system to investigate the prognostic effect of positive peritoneal cytology. All five pointed in the same direction and suggested that positive peritoneal cytology is not an independent prognostic factor in endometrial cancer, that is, some studies showed no survival difference between stage I and cytological stage IIIA cancer (Grimshaw *et al*, 1990; Kadar *et al*, 1992; Kasamatsu *et al*, 2003; Tebeu *et al*, 2003) and others showed an important survival difference within stage IIIA patients, that is, significantly better prognosis of cytological stage IIIA patients over histological stage IIIA patients (Preyer *et al*, 2002; Tebeu *et al*, 2003). These studies had several shortcomings. Several were not able to adjust for use of radiotherapy, making it difficult to estimate the effect of peritoneal cytology in itself (Grimshaw *et al*, 1990; Kadar *et al*, 1992; Preyer *et al*, 2002; Tebeu *et al*, 2003). Some studies included patients with cervical involvement, but did not adjust for the possible impact of this prognostic factor (Kadar *et al*, 1992; Preyer *et al*, 2002; Kasamatsu *et al*, 2003).

In the present study, we have eliminated most of the problems of the other studies. We included stage I and both cytological and histological stage IIIA patients. We used surgical staging, which enabled us to adjust for prognostic factors such as grade and myometrial invasion. In addition, we included both patients with and without radiotherapy and we were able to adjust for this variable as well. In addition, patients with cervical invasion were excluded, making the results easy to interpret. There is no survival difference between patients with stage I endometrial cancer and stage IIIA endometrial cancer based on positive peritoneal cytology alone, even after adjusting for other prognostic factors. This strongly suggests that positive peritoneal cytology is not an independent prognostic factor in patients with endometrial cancer.

We acknowledge that our study suffered from a relatively low statistical power due to the limited number of patients with cytological and histological stage IIIA endometrial cancer. In particular, subgroup analyses according to the use of radiotherapy were based on low patient numbers, and we therefore cannot give a definitive conclusion on the lack of benefit of radiotherapy among early endometrial cancer patients with positive peritoneal cytology. Additional research on the usefulness of radiotherapy for this particular patient category might be indicated. Nevertheless, this study confirms previous studies and strongly suggests that the 1988 FIGO classification system classifies patients with cancer confined to the uterus and with positive peritoneal cytology alone into an inappropriate risk category. Owing to the present and previous studies, FIGO might consider reviewing its 1988 classification system.

## References

[bib3] AJCC (2002) AJCC Cancer Staging Handbook. From the AJCC Cancer Staging Manual. New York: Springer

[bib4] Bouchardy C (1997) Switzerland, Geneva. In Cancer Incidence in Five Continents, Vol. VII Parkin DM, Whelan SL, Ferlay J, Raymond L, Young J (eds) pp 666–669, Lyon: International Agency for Research on Cancer

[bib5] Burke TW, Eifel PJ, Muggia FM (1997) Cancer of the uterine body. In Cancer Principles and Pratice of Oncology, DeVita Jr VT, Hellman S, Rosenberg SA (eds) pp 1478–1499, Philadelphia, NY: Lippincott-Raven

[bib6] Creasman WT, Disaia PJ, Blessing J, Wilkinson Jr RH, Johnston W, Weed Jr JC (1981) Prognostic significance of peritoneal cytology in patients with endometrial cancer and preliminary data concerning therapy with intraperitoneal radiopharmaceuticals. Am J Obstet Gynecol 141: 921–929 7315922 10.1016/s0002-9378(16)32684-9

[bib7] Creasman WT, Morrow CP, Bundy BN, Homesley HD, Graham JE, Heller PB (1987) Surgical pathologic spread patterns of endometrial cancer. A Gynecologic Oncology Group Study. Cancer 60: 2035–2041 3652025 10.1002/1097-0142(19901015)60:8+<2035::aid-cncr2820601515>3.0.co;2-8

[bib2] FIGO (2000) Staging Classifications and Clinical Practice Guidelines of Gynecological Cancers by the FIGO Committee on Gynecologic Oncology. Oxford: Elsevier 11041682

[bib8] Grady D, Ernster VL (1996) Endometrial cancer. In Cancer Epidemiology and Prevention, Schottenfeld D, Fraumeni Jr JF (eds) pp 1058–1089, New York: Oxford University Press

[bib9] Grimshaw RN, Tupper WC, Fraser RC, Tompkins MG, Jeffrey JF (1990) Prognostic value of peritoneal cytology in endometrial carcinoma. Gynecol Oncol 36: 97–100 2295459 10.1016/0090-8258(90)90116-3

[bib10] Harouny VR, Sutton GP, Clark SA, Geisler HE, Stehman FB, Ehrlich CE (1988) The importance of peritoneal cytology in endometrial carcinoma. Obstet Gynecol 72: 394–398 3405555

[bib11] Hull CH, Nie NH (1995) SPSS Update 7–9. New Procedures for Releases 7–9. New York: McGraw-Hill Book Company

[bib12] Ide P (1984) Prognostic value of peritoneal fluid cytology in patients with endometrial cancer stage I. Eur J Obstet Gynecol Reprod Biol 18: 343–349 6526121 10.1016/0028-2243(84)90056-x

[bib13] Kadar N, Homesley HD, Malfetano JH (1992) Positive peritoneal cytology is an adverse factor in endometrial carcinoma only if there is other evidence of extrauterine disease. Gynecol Oncol 46: 145–149 1500014 10.1016/0090-8258(92)90246-f

[bib14] Kasamatsu T, Onda T, Katsumata N, Sawada M, Yamada T, Tsunematsu R, Ohmi K, Sasajima Y, Matsuno Y (2003) Prognostic significance of positive peritoneal cytology in endometrial carcinoma confined to the uterus. Br J Cancer 88: 245–250 12610496 10.1038/sj.bjc.6600698 PMC2377042

[bib15] Konski A, Poulter C, Keys H, Rubin P, Beecham J, Doane K (1988) Absence of prognostic significance, peritoneal dissemination and treatment advantage in endometrial cancer patients with positive peritoneal cytology. Int J Radiat Oncol Biol Phys 14: 49–55 3335462 10.1016/0360-3016(88)90050-8

[bib16] Morrow CP, Bundy BN, Kurman RJ, Creasman WT, Heller P, Homesley HD, Graham JE (1991) Relationship between surgical-pathological risk factors and outcome in clinical stage I and II carcinoma of the endometrium: a Gynecologic Oncology Group Study. Gynecol Oncol 40: 55–65 1989916 10.1016/0090-8258(91)90086-k

[bib17] Obermair A, Geramou M, Tripcony L, Nicklin JL, Perrin L, Crandon AJ (2001) Peritoneal cytology: impact on disease-free survival in clinical stage I endometroid adenocarcinoma of the uterus. Cancer Lett 164: 105–110 11166922 10.1016/s0304-3835(00)00722-9

[bib18] Preyer O, Obermair A, Formann E, Schmid W, Perrin LC, Ward BG, Crandon AJ, Nicklin JL (2002) The impact of positive peritoneal washings and serosal and adnexal involvement on survival in patients with stage IIIA uterine cancer. Gynecol Oncol 86: 269–273 12217747 10.1006/gyno.2002.6744

[bib19] Tebeu PM, Popowski GY, Verkooijen HM, Casals J, Ludicke F, Zeciri G, Usel M, Bouchardy C, Major AL (2003) Impact of peritoneal cytology on survival of endometrial cancer patients treated with surgery and radiotherapy. Br J Cancer 89: 2023–2026 14647132 10.1038/sj.bjc.6601446 PMC2376850

[bib1] WHO (1976) ICD-O International Classification of Diseases for Oncology, 1st edn Geneva: World Health Organization

